# From Synergy to Complexity: The Trend Toward Integrated Value Chain and Landscape Governance

**DOI:** 10.1007/s00267-018-1055-0

**Published:** 2018-05-30

**Authors:** Mirjam A. F. Ros-Tonen, James Reed, Terry Sunderland

**Affiliations:** 1 Department of Geography, Planning and International Development Studies and Centre for Sustainable Development Studies, University of Amsterdam, P.O. Box 15629, 1001 NC Amsterdam, The Netherlands; 2 Center for International Forestry Research (CIFOR) Jalan CIFOR, Situ Gede, 16115 Bogor, West Java Indonesia; 3 University of British Columbia (UBC) and Center for International Forestry Research (CIFOR), Forest Sciences Centre 2424 Main Mall Vancouver, BC, V6T 1Z4 Canada

**Keywords:** Integrated landscape approaches, Integrated landscape-level initiatives, Landscape governance, Institutions, Multi-stakeholder platforms, Bridging actors

## Abstract

This Editorial introduces a special issue that illustrates a trend toward integrated landscape approaches. Whereas two papers echo older “win–win” strategies based on the trade of non-timber forest products, ten papers reflect a shift from a product to landscape perspective. However, they differ from integrated landscape approaches in that they emanate from sectorial approaches driven primarily by aims such as forest restoration, sustainable commodity sourcing, natural resource management, or carbon emission reduction. The potential of such initiatives for integrated landscape governance and achieving landscape-level outcomes has hitherto been largely unaddressed in the literature on integrated landscape approaches. This special issue addresses this gap, with a focus on actor constellations and institutional arrangements emerging in the transition from sectorial to integrated approaches. This editorial discusses the trends arising from the papers, including the need for a commonly shared concern and sense of urgency; inclusive stakeholder engagement; accommodating and coordinating polycentric governance in landscapes beset with institutional fragmentation and jurisdictional mismatches; alignment with locally embedded initiatives and governance structures; and a framework to assess and monitor the performance of integrated multi-stakeholder approaches. We conclude that, despite a growing tendency toward integrated approaches at the landscape level, inherent landscape complexity renders persistent and significant challenges such as balancing multiple objectives, equitable inclusion of all relevant stakeholders, dealing with power and gender asymmetries, adaptive management based on participatory outcome monitoring, and moving beyond existing administrative, jurisdictional, and sectorial silos. Multi-stakeholder platforms and bridging organizations and individuals are seen as key in overcoming such challenges.

## Introduction

Integrated landscape approaches (ILAs) or initiatives (ILIs)^1^ have been promoted by a broad range of international conservation and development organizations^2^ as a governance approach to reconcile local-global challenges such as biodiversity loss, climate change, food insecurity, and poverty at the landscape level (Harvey et al. 2008; Scherr et al. 2012; Harvey et al. 2014; Kozar et al. 2014; Padoch and Sunderland 2014; Reed et al. 2015, 2016). ILAs represent the most recent attempt to reconcile conservation and development objectives, following on from Integrated Conservation and Development Projects (ICDPs) in the 1980s and strategies embarking on non-timber forest product (NTFP) trade in the 1990s (Reed et al. 2017). In contrast with the earlier approaches, ILAs recognize that such problems cannot be addressed in isolation and that, at the landscape level, tackling one problem invariably involves trade-offs with another (Sunderland et al. 2008). ILAs therefore call for solutions based on a common concern entry point and change logic negotiated in multi-stakeholder settings, characterized by multifunctionality, multiple scales,^3^flexibility, adaptive management and continual learning (Sayer et al. 2013; Ros-Tonen et al. 2014; Freeman et al. 2015; Sayer et al. 2015). Implementation in practice results in new actor constellations, and hybrid and polycentric institutional arrangements involving multiple centers of decision-making (Nagendra and Ostrom 2012) that challenge existing sectorial, administrative and jurisdictional boundaries (van Oosten 2013; Ros-Tonen et al. 2015a; Visseren-Hamakers 2015). This requires a more nuanced understanding of the diversity of governance arrangements within complex landscapes, and the ability to better align actor aspirations and needs across multiple levels in landscape governance. We thereby distinguish landscape governance from landscape approaches, using the latter as a general denominator for processes, tools, and concepts for allocating and managing land within a landscape of competing land uses (Sayer et al. 2013, p. 8349) and “landscape governance” to denote the more general process of steering human–nature interactions in a bounded geographical space (*c.f.* Görg 2007; van Oosten et al. 2014; Ros-Tonen et al. 2014; van Oosten et al. 2018; Rodríguez-Ward et al. 2018).

There is a great variety in how landscape approaches are conceptualized and labeled (Pfund 2010; Reed et al. 2016; Sayer et al. 2013; Scherr et al. 2013; Erbaugh and Agrawal 2017). A recent distinction has been made between ILAs as cross-sectorial landscape approaches that make a deliberate effort to “achieve multiple functional goals through collective action and integrated governance”, and integrated landscape-scale initiatives that tend to focus on a primary goal around a defined social, ecological, or political boundary (Zanzanaini et al. 2017, p. 12; see also Kozar et al. 2014; Kusters et al. 2018, this issue). Most cases analyzed in this special issue concern the latter: such initiatives qualify as integrated approaches for tackling multiple aims in multifunctional landscapes with multi-stakeholder involvement, but emanate from sectorial approaches that are driven by an underlying primary aim such as forest and landscape restoration (Eriksson et al. 2018; Foli et al. 2018; Long et al. 2018, this issue); sustainable natural resource management (Dale et al. 2018; Foli et al. 2018, this issue); carbon emission reduction (Brown 2018; Rodríguez-Ward et al. 2018, this issue); or sustainable sourcing of commodities (Deans et al. 2018; Ingram et al. 2018; van Oosten et al. 2018, this issue; Table 1). Consistent with the distinction between scale and level (footnote 3), we refer to such initiatives as integrated landscape-level initiatives (ILLIs). However, little is known about the potential of such initiatives to contribute to integrated landscape governance, for which they were not designed in the first place. This is the first knowledge gap that this special issue addresses. Second, most integrated approaches are still largely experimental, and their institutional arrangements and overall effectiveness remain relatively under-researched (Reed et al. 2017). This makes research into the nature and inclusiveness of institutional arrangements that govern integrated approaches opportune. Third, the scientific literature has hitherto focused mainly on the design principles and frameworks of ILAs (Sayer et al. 2013; Ros-Tonen et al. 2014; Freeman et al. 2015; Bürgi et al. 2017) and the documentation of case studies (Estrada-Carmona et al. 2014; Milder et al. 2014; Minang et al. 2015; NAS 2016; García Martín et al. 2016; Zanzanaini et al. 2017). Much less is known about how ILAs should be operationalized over the longer term and how landscape-level governance can be aligned with existing institutional frameworks. This special issue attempts to address these gaps, with a focus on institutional arrangements and actor constellations emerging in the transition from sectorial to integrated approaches. On the basis of empirical case studies carried out mostly in forested and tree-dominated landscapes, the papers shed light on how sectorial policies are being redesigned toward more integrated approaches; sometimes as a reverberation of the earlier NTFP debate (Lowore et al. 2018 and Ndeidoma et al. 2018, this issue), but mostly as new integrated approaches targeting the landscape level. The studies examine how the tendency toward such approaches creates synergies with, or challenges existing jurisdictions in, for instance, integrated forest and water management, natural resource management schemes, climate change mitigation, and sustainable value chain governance. In doing so, this volume explores whether and how existing institutions within the landscape can be merged into new governance configurations for greater synergy in achieving landscape-level outcomes. As such, this collection of papers contributes to topical, and sometime contentious, debates on landscape governance, integrated natural resource management, and value chain governance.

**Table 1:** Overview of integrated landscape-level initiatives (ILLIs) analyzed in this issue^a^

Integrated landscape-level initiatives emanating from sectorial approaches
**Forest and landscape restoration**
1. Integrated forest and water management, Sweden (Eriksson et al. 2018)
2. Forest restoration, China (Long et al. 2018)
3. Reforestation through co-management (MTS), Ghana (Foli et al. 2018)
**Natural resource management schemes**
4. Great Barrier Reef, Australia (Dale et al. 2018)
5. Community resource management (CREMA), Ghana (Foli et al. 2018)
6. Chantier d’Aménagment Forestier (CAF), Burkina Faso (Foli et al. 2018)
**Climate change mitigation**
7. REDD+, Peru (Rodríguez-Ward et al. 2018)
8. REDD+, Cameroon (Brown 2018)
**Sustainable value chain governance**
9. Value chain governance for environmental services, The Netherlands (Ingram et al. 2018)
10. Value chain collaboration, Ghana (Deans et al. 2018)
11. Oil palm public–private partnership, Indonesia (van Oosten et al. 2018)

^a^The papers by Lowore et al. (2018) and Ndeinoma et al. (2018) are excluded from this overview as they deal with “win–win” strategies based on the trade of non-timber forest products, without targeting the landscape level. Kusters et al. (2018) is excluded from this table as the paper refers to a method designed for integrated landscape approaches from the beginning

*MTS* modified taungya system, *CREMA* community resource management area, *CAF* Chantier d’Aménagement Forestier, *REDD*+ reducing emissions from deforestation and forest degradation

Building upon recent literature on ILAs and drawing from the contributions to this volume, this editorial first provides an overview of ILAs and how they differ from earlier approaches such as ICDPs and NTFP strategies that aimed to reconcile multiple aims. After this initial focus on ILAs, we move to ILLIs in areas such as forest and landscape restoration (Eriksson et al. 2018; Long et al. 2018; Foli et al. 2018, this issue); natural resource management (Dale et al. 2018; Foli et al. 2018, this issue); climate change mitigation (Brown 2018; Rodríguez-Ward et al. 2018, this issue); and value chain governance (Deans et al. 2018; Ingram et al. 2018; van Oosten et al. 2018, this issue) that can potentially be aligned with ILAs. Considering the significant institutional challenges of operating beyond sectors, we thereby, respectively, address the need for a commonly shared concern and sense of urgency; multi-stakeholder platforms, bridging organizations, and “multilevel hybrids” (Rodríguez-Ward et al. 2018; Brown 2018, this issue); private sector engagement; dealing with conflicting interests and power imbalances; accommodating and coordinating polycentric governance in landscapes beset with institutional fragmentation and jurisdictional mismatches; alignment with locally embedded initiatives and governance structures; and a framework to assess and monitor performance of integrated multi-stakeholder approaches. The papers herein collectively conclude that there is a growing tendency toward integrated approaches and creating synergies at landscape level, but that “muddling through” (Lindblom 1959; Sayer et al. 2008; Colfer et al. 2011) is a fundamental characteristic of such initiatives due to the inherent dynamism of landscapes and the actors and activities associated with them (Sayer et al. 2016).

### **From “Win–Wins” to “Winning More and Losing Less”**

Integrated landscape approaches represent the latest in a series of attempts to reconcile issues affecting society and environment at multiple scales. Previous attempts, like promoting the trade in NTFPs, were based on the notion of delivering “win–win” outcomes, i.e., both positive social and environmental impacts. Despite this intention to reconcile multiple aims (livelihood improvement and nature conservation), the strategy was based on a sectorial focus on one or a few NTFPs, hardly considering trade-offs with other land uses, activities, or actor interests (Ros-Tonen and Wiersum 2005; Sunderland et al. 2008). This is reflected in the article on forest honey in Cameroon by Lowore et al. (2018, this issue), which we included in this special issue to illustrate how thinking about integrated objectives has evolved from a product to landscape focus. Although representative for the rather naive belief in “win–wins” through NTFP trade, the authors note the importance of partnerships to achieve objectives to scale and of taking past and present land uses into account.

The need to ensure and coordinate broad stakeholder involvement as well as learning processes to achieve such “win–win” outcomes is addressed in the paper on NTFP governance in Namibia (Ndeinoma et al. 2018, this issue). Using a policy network analysis, the authors identify the Indigenous Plant Task Team (IPTT) as a key actor in the governance of what in Namibia is labeled as “Indigenous Natural Products”. The IPTT was established as a multistakeholder forum to mobilize actors, funds, and knowledge, as well as to design policies and coordinate the development of products and markets. It eventually turned into an organization in which the interests of government actors and consultants prevailed, and in which traders, small-scale enterprises, harvesters, and community-based organizations are hardly, if at all, involved. Despite its policy influence, this has resulted in a neglect of local-level interests in policy formulation and implementation. It also illustrates the difficulty of achieving inclusive governance structures in “win–win” strategies and landscape approaches more broadly.

However, what these NTFP strategies (and, earlier, ICDPs) are particularly lacking is the recognition that not all stakeholders will “win” all of the time (Sunderland et al. 2013). Therefore, landscape approaches—in theory at least—recognize the inherent dynamism of landscapes and are built on the premise of the need for overall facilitation of stakeholder negotiation, trade-off analysis, and processes of adaptive management to ensure that stakeholders are “winning more and losing less” (Sayer et al. 2013). Through independently facilitated platforms that acknowledge the diversity of stakeholder needs, objectives, and power positions, potential synergies and trade-offs can be identified in order to develop a shared vision for the management of a wider landscape. With regular and ongoing negotiation, processes of adaptive management can be implemented and continually reflected upon to enhance synergies and seek alternative implementation strategies where major trade-offs occur (Sunderland et al. 2008; Sayer et al. 2015).

There is a growing body of work that provides evidence of uptake of ILAs. One of these is a global review undertaken by EcoAgriculture Partners and collaborators—primarily based on grass-roots perspectives and using standardized questionnaire methods—in Latin America (Estrada-Carmona et al. 2014), Africa (Milder et al. 2014), Europe (García Martín et al. 2016), and Asia (Zanzanaini et al. 2017). This review showed that local stakeholders reported successful integrated conservation and development outcomes consistently across the three continents. A review of peer-reviewed evidence provides further—although limited—support for the effectiveness of ILAs in practice (Reed et al. 2017). Finally, the abundant body of literature detailing the assumed characteristics of ILAs (Sayer et al. 2013; Ros-Tonen et al. 2014; Minang et al. 2015; Freeman et al. 2015; Reed et al. 2017) demonstrates a real distinction between ILAs and previous attempts at integration in moving from a primary focus on biodiversity conservation and local development alone (McShane and Wells 2004) to broader land-use issues.

While there has been increasing support for ILAs, the approach has also been questioned for its utility and viability, notably compared to territorial approaches toward indigenous and local community management of natural resources (McCall 2016). Recent discourse has also suggested that the uptake of landscape approaches in practice is slow, at best (Reed et al. 2017) and that the approach may merely be the latest conservation and development fad (Redford et al. 2013; Lund et al. 2017), a re-branding of previous efforts (Redford et al. 2013; Reed et al. 2017; Bastos Lima et al. 2017) or a “new management ethic” (Erbaugh and Agrawal 2017). This is related to a number of challenges that prevent ILAs from moving beyond their obvious theoretical potential. First, ILAs face challenges in the operationalization of the approach on the ground (Freeman et al. 2015; Bürgi et al. 2017). These challenges often relate to the scale at which implementation and maintenance of an ILA take place—both of a spatial and temporal nature. Second, it has been recognized that landscapes do not coincide with either jurisdictional boundaries (Kozar et al. 2014; Ros-Tonen et al. 2014; Arts et al. 2017) or territorial boundaries (McCall 2016) and may therefore face administrative impediments to implementation. Third, ILAs have been criticized for downplaying power imbalances (Lebel and Daniel 2009; Clay 2016; Arts et al. 2017) and considering landscapes as depoliticized spaces (Müller et al. 2015). Fourth, ILAs face challenges to monitor and evaluate impact due to their nature as a long-term process and the need to account for trade-offs (Sunderland et al. 2008; Lebel and Daniel 2009; Sayer et al. 2015; Sayer et al. 2016). Fifth, due to their ambitions, ILAs require considerable funds and time investments to involve all stakeholders (Reed et al. 2016; Bürgi et al. 2017). In this special issue we therefore look at the potential to address these issues through landscape-level initiatives that were initially designed as sectorial approaches, but which are moving toward increasing integration of objectives and stakeholder involvement to achieve sustainable multifunctional landscapes.

### The Basis of Integrated Approaches: Shared Concerns and Sense of Urgency

An integrated approach begins with a “common concern entry point” (Sayer et al. 2013) or “value proposition” (Sayer et al. 2015). As with any multi-stakeholder approach, actors are likely to join the process only if there is a commonly felt problem and sense of urgency to act upon it. As regional overviews of ILAs have demonstrated, over 70% of landscape initiatives are primarily driven by conservation and restoration motives, but also that an increasing proportion is situated in multifunctional landscapes where they seek to balance production with conservation and sustainability aims (Estrada-Carmona et al. 2014; Milder et al. 2014; García-Martín et al. 2016; Sayer et al. 2016; Zanzanaini et al. 2017). As outlined in the introduction, the latter also applies to the case studies brought together in this issue with resource depletion, deforestation, and environmental degradation being the primary drivers of landscape-level initiatives, and livelihood concerns and water management often being the secondary concerns (Table 2). As the paper by Dale et al. (2018, this issue) illustrates, the lack of a sense of urgency (in this case of ensuring water quality) impedes an integrated approach at the catchment landscape level. Similarly, the Ghana pilot described in Kusters et al. (2018, this issue) revealed that follow-up on a workshop that brought stakeholders together to discuss collaboration at the landscape level remained “in the air” because of the lack of a commonly felt sense of urgency around a particular issue. Contrastingly, general awareness of the need to combine forest restoration with water resource management was at the basis of landscape-level initiatives in Sweden (Eriksson et al. 2018, this issue).

**Table 2:** Shared concerns in the cases of this special issue

Case study	Primary concern	Secondary concerns
African forest honey, Ethiopia (Lowore et al. 2018)	Livelihood improvement; trade development	Forest conservation
Non-timber forest products (NTFPs), Namibia (Ndeinoma et al. 2018)	Improve production and marketing of NTFPs; enhance coordination (resource mobilization and knowledge exchange) among actors	Livelihood improvement
Integrated forest and water management, Sweden (Eriksson et al. 2018)	Forest restoration and sustainable forest management	Water management, preserving environmental and social values, climate change mitigation and adaptation
Ecological Forest Purchase Program, China (Long et al. 2018)	Forest landscape restoration and safeguarding the provision of environmental services	Improving rural livelihoods through compensatory payments
Great Barrier Reef, Australia (Dale et al. 2018)	Various depending on the governance subdomain (ecological health of water flows, natural resource management, land-use planning, farm productivity, indigenous management)	Water quality (but sense of urgency missing and hence mostly neglected at catchment landscape level)
Reforestation through co-management (MTS), Ghana (Foli et al. 2018)	Future timber supply; forest restoration	Livelihood improvement through interplanting food crops and sharing in timber benefits
Community resource management (CREMA) and Chantier d’Aménagment Forestier (CAF), Burkina Faso, Ghana (Foli et al. 2018)	Natural resource management	Livelihood improvement
REDD+, Peru (Rodríguez-Ward et al. 2018)	Reducing carbon emissions from deforestation and forest degradation/climate change mitigation	Biodiversity conservation, land-use planning to reconcile competing land uses (e.g., mining, farming, Brazil nut extraction)
REDD+, Cameroon (Brown 2018)	Climate change mitigation	Forest conservation, livelihood improvement
Value chain governance for environmental services, the Netherlands (Ingram et al. 2018)	Sustainable sourcing of several commodities	Conservation of environmental services
Value chain collaboration, Ghana (Deans et al. 2018)	Sustainable sourcing of cocoa—water and biodiversity conservation, pollution control, waste management, preventing supplier failure	Livelihood improvement through input provision and training, increasing climate resilience
Multifunctional oil palm concession, Indonesia (van Oosten et al. 2018)	Sustainable sourcing of palm oil—protection of riparian zones and high conservation value forests; meeting Zero Deforestation pledges^a^	Increasing the inclusiveness of the company’s production model through a collaborative landscape design that integrates smallholder land uses (e.g., rubber agro-forests, cultural–spiritual sites).
Multi-stakeholder platforms, Ghana and Indonesia (Kusters et al. 2018)	Explore options for landscape-level multi-stakeholder processes and outcomes	—

^a^The Zero Deforestation movement emanated from the non-binding New York Declaration on Forests (2014) and comprises a private sector commitment to eliminate deforestation from agricultural commodities (http://forestdeclaration.org/, accessed 3 January 2018)

## Actor Constellations

### The Importance of Multi-stakeholder Platforms, Bridging Organizations and “Multilevel Hybrids”

Evidence in this special issue suggests that hybrid, multi-level governance arrangements that marry top-down authoritarian processes with more bottom-up democratic structures may be optimal for continued engagement in, and effectiveness of, ILAs and ILLIs (Long et al. 2018; Ingram et al. 2018; Rodriguez-Ward et al. 2018). How such governance arrangements are operationalized will be both dependent upon—and influenced by—local context and existing social arrangements, depending on the place-based interactions between natural conditions and the political economy (van Oosten et al. 2018); whether more or less formalized governance systems are in place (Dale et al. 2018; Long et al. 2018; Foli et al. 2018); previous experiences with collaboration, positive or negative (conflicts, mistrust; Rodríguez-Ward et al. 2018); the degree to which the private sector or bridging organizations such as NGOs or research organizations play a role (Deans et al. 2018; Ingram et al. 2018; Kusters et al. 2018; Eriksson et al. 2018); and the financial capacity of the organizations involved (Ndeinoma et al. 2018; Brown 2018, this issue).

Theoretically, such an arrangement should feature a platform that provides an enabling environment for local actors and broader stakeholders with an interest in the landscape to regularly—or at least intermittently—negotiate objectives and exchange desired outcomes (Kusters et al. 2018). In practice, maintaining such a platform is often fraught with difficulty and identifying leverage points to best overcome challenges will be fundamental to maintain long-term engagement from a diverse group of stakeholders. Such leverage points may be initially underestimated, but overlooking them could disproportionately affect the efficiency of adaptive governance processes and perpetuate marginalization of local stakeholders, as illustrated in the paper by Ndeinoma et al. (2018). Examples include identifying an appropriate location such that all concerned stakeholders have the capacity to attend; ensuring that opportunities to engage in such platforms are equitable; providing independent facilitation to build trust among attendees; enhancing transparency via monitoring of the efficiency of the platform and dissemination of results and progress; and embracing, rather than avoiding, issues that may be anticipated to lead to social or environmental conflicts (Kusters et al. 2018; see also Sayer et al. 2015; Bürgi et al. 2017).

Actors capable of bridging different sectors and levels play an important role in initiating and maintaining such platforms. Such actors function as bridging organizations and can be a research organization, NGO, “ecomuseum” (Hahn et al. 2006) or an agro-ecological partnership (Prager 2015). They mobilize actors, funds, and political support; broker information and different knowledges; build trust and social capital; mediate conflicts; network and communicate across scales; facilitate linkages between different actors; and create platforms for collective learning (Folke et al. 2005; Cash et al. 2006; Hahn et al. 2006; Berkes 2009; Leys and Vanclay 2011; Rathwell and Peterson 2012; Crona and Parker 2012; Ros-Tonen et al. 2014; Ros-Tonen et al. 2015b; Prager 2015). Examples from the papers in this issue include the Stockholm Water Institute that mobilized stakeholders for integrated forest and water management (Eriksson et al. 2018); the State-led Yong’an Volunteer Association for Promoting Ecological Civilization that mobilized funds and actors to implement a forest landscape restoration program in a municipality in China (Long et al. 2018); the World Wide Fund for Nature (WWF), which mobilized actors and funds to implement REDD+ in Cameroon (Brown 2018); and the NGOs (Tropenbos International and EcoAgriculture Partners) that piloted the platform methodology in Ghana and Indonesia (Kusters et al. 2018). The Indigenous Plant Task Team in Namibia was established to perform such a role for the country’s NTFP sector (Ndeinoma et al. 2018). Rodríguez-Ward et al. (2018) point to a particular bridging actor, to which they refer as “multilevel hybrids”: individuals who navigate across levels and sectors and act as binding factors between those. In the REDD+ case in Cameroon, the WWF was identified as such a hybrid (Brown, 2018).

Proponents of ILAs recommend that implementation should focus on establishing a multi-stakeholder platform from the outset (Sayer et al. 2013; Denier et al. 2015; Reed et al. 2016). By bringing together those with a vested interest in the landscape of interest, individual and collective needs, objectives and trade-offs can be identified and management be applied accordingly. In theory, this should help to dissolve power asymmetries and ensure that local and marginalized groups can express what it is they hope for, rather than what it is they are willing to accept. In practical terms, it has to be questioned how realistic such a scenario is likely to be. Recent experience of ILAs being implemented suggests that more often the case is that an implementing organization enters with a pre-conceived set of criteria that they wish to fulfill (Clay 2016; Weatherley-Singh and Gupta 2017). This, however, need not diminish the potential use, and value, of ILA principles—even when objectives are pre-defined, the efficacy of the intervention will likely be heightened with regular stakeholder negotiation that seeks to enhance local capacity and increase landscape multifunctionality and resilience.

### The Role of the Private Sector

Earlier reviews of mainly conservation-induced ILAs revealed limited participation of the private sector, with averages between 10 and 14% (*N* = 87) for Africa and 22% (local agribusinesses) and 7% (foreign agribusinesses; *N* = 104) for Latin America and the Caribbean (Milder et al. 2014; Estrada-Carmona et al. 2014; Hart et al. 2015). The picture is definitively different for the ILLIs reported in this special issue, with the private sector participating in almost all (95.8%) of the 24 cases^4^ and taking the lead in 41.7% of them.^5^ Companies’ main motivations to engage in ILLIs are related to sustainable sourcing: to prevent supply failure in the future, reduce the ecological impact in sourcing areas, and/or to satisfy consumers’ and NGO demands for sustainably sourced products and benefit from price premiums on markets for certified products (Deans et al. 2018; van Oosten et al. 2018; Ingram et al. 2018; Eriksson et al. 2018). These reasons resonate with those mentioned by Arts et al. (2017) regarding the attractiveness of landscape approaches for the private sector, to which they add the opportunity for innovations by promoting improved farming methods and increased product quality. Such innovations can also be found in this special issue. One is the multifunctional oil palm concession in Indonesia, which combines commodity production with the protection of riparian zones, high conservation forests, and —important for local communities—multifunctional rubber gardens and cultural–spiritual sites (van Oosten et al. 2018). Another is the inclusion of payments for environmental services (PES) schemes in the cocoa trade (Ingram et al. 2018). A secondary motivation is often to increase the wellbeing of producers, particularly where youth needs to be attracted to replace an ageing farmer population such as in Ghana’s cocoa sector (Deans et al. 2018).

As made clear in this special issue, private sector-initiated ILLIs require new actor configurations and institutional arrangements (van Oosten et al. 2018) that go “beyond the farm” (Deans et al. 2018), “beyond the chain” (Ros-Tonen et al. 2015; Deans et al. 2018), and “beyond certification” (Ingram et al. 2018). This would entail greater synergies between the agricultural, forestry, and environmental sectors (in terms of policies, laws, and actors); between public and private governance (e.g., by combining procurement policies with certification); and between governance levels (global to local). Public–private partnerships and landscape-level certification are avenues to achieving such synergies (Deans et al. 2018; Ingram et al. 2018; van Oosten et al. 2018).

Where such alliances can be created and value chain and landscape governance can be linked (Ingram et al. 2018), there is a great potential for implementing landscape approaches at the producer end of value chains (see also section on locally embedded entry points). This implies a potentially important and even key role for the private sector in the implementation of landscape approaches (Sayer et al. 2015; see also Wambugu et al. 2015; Kissinger et al. 2015; Gyau et al. 2015) and in increasing the economic sustainability of such initiatives (Deans et al. 2018). Some scholars, however, question the inclusiveness of private sector-led arrangements and the equitability of risk and benefit sharing, and point at risks such as a narrow commodity focus, privatization of natural resources, greenwashing, and outcompeting small producers (Namirembe and Bernard 2015; Ros-Tonen et al. 2015a; Arts et al. 2017). This brings us to the broader challenge of dealing with power imbalances in multi-stakeholder processes.

### The Challenge of Dealing with Power Imbalances

Landscapes with non-administrative boundaries might poorly reflect the rights and requirements of local actors. However, thinking beyond jurisdictional boundaries (see next section) and the dichotomy of top-down vs. bottom-up governance will likely be necessary to better account for the multiple conflicting processes occurring across tropical developing landscapes. ILAs need not be considered a departure from community-based conservation (Berkes 2009) or rights-based development approaches (Johnson and Forsyth 2002; Campese et al. 2009; Reed et al. 2017; Erbaugh and Agrawal 2017). Indeed, the key principles of ILAs as defined by Sayer et al. (2013) are clear recognition of rights and responsibilities and common concern entry points. An ILA attempts to incorporate these values within broader landscape processes, identify pressures and feedback mechanisms, account for inevitable trade-offs, and internalize potential externalities. However, ILLIs such as those reviewed in this special issue were not primarily designed as ILAs and may therefore pose challenges to dealing with power imbalances and giving a voice to marginalized people, as do the “win–win” strategies in Namibia’s NTFP sector (Ndeinoma et al. 2018). The paper by Rodríguez-Ward et al. (2018) illustrates this well, demonstrating that particular land-user associations (farmers and miners) were largely excluded from the REDD+ process in Madre de Dios, Peru. Similarly, Deans et al. (2018), within a context of value chain collaboration for certified cocoa production in Ghana, note that the company invested considerably in the relationship with farmers, but that there was limited space for farmers to negotiate their interests further down the value chain. In China, space was given to citizen participation in forest landscape restoration, but local citizens were marginalized from decision-making and monitoring (Long et al. 2018). The issue of adequate and equitable stakeholder representation and voice is not typical for landscape-level initiatives, but inherent in any multi-stakeholder initiative in situations marked by power imbalances and unequal access to natural resources.

Evidence from the conservation literature has shown that strictly bottom-up approaches can produce perverse outcomes (Carpentier et al. 1999; Wunder 2001; Brown 2002), whereas local stakeholders may lack the necessary skills to effectively negotiate and compromise in multi-stakeholder dialogs (Hemmati 2002; Reed et al. 2015). Meanwhile, interventions that are constrained by jurisdictional boundaries may represent an opportunity to better influence local policy formulation (Rodríguez-Ward et al. 2018; van Oosten et al. 2018), but are also be subject to leakage effects in neighboring jurisdictions (Atmadja and Verchot 2012; Carrasco et al. 2017). Similarly, commodity supply chains typically operate across jurisdictional and national boundaries, but are often of a hierarchical nature, leaving local stakeholders vulnerable to inadequate representation in negotiation processes and making regulation and traceability particularly challenging (Ndeinoma et al. 2018; Deans et al. 2018). Multi-stakeholder dialog platforms have been suggested as a solution to create space for negotiation and knowledge exchange (Cullen et al. 2014; Ros-Tonen et al. 2015a; Ndeinoma et al. 2018; Kusters et al. 2018), but evidence from the water sector has shown that little power sharing (notably vertical inclusion) takes place in such platforms, while some actors may strategically withhold knowledge (Warner 2006). Similar experiences were observed in the platforms piloted in Ghana and Indonesia by Kusters et al. (2018). As with all multisectorial partnerships and multi-stakeholder processes this requires third-party brokers as “watchdogs” to defend the interests of the weaker parties (Mayers and Vermeulen 2002; Ros-Tonen et al. 2008; Dale et al. 2018). These may be NGOs or political alliances, but also the active involvement of the State, including at subnational levels, is crucial in this respect (Wambugu et al. 2015; Kissinger et al. 2015; Ndeinoma et al. 2018; Rodríguez-Ward et al. 2018).

### Institutional Synergies and Jurisdictional Mismatches: The Need for Accommodating and Coordinating Polycentric Governance

Implementing landscape-level initiatives must confront the challenge of institutional and jurisdictional fragmentation and institutional rigidity, whereby in particular public actors adhere to their sectorial silos and jurisdictional powers (van Oosten et al. 2013; van Oosten et al. 2018; Long et al. 2018; Rodríguez-Ward 2018, this issue). This mismatch between landscapes and existing administrative jurisdictions implies the necessity of dealing with multiple centers of decision-making that operate at different scale levels (Dale et al. 2018). “Polycentric governance” has been proposed to cover such different, but partly overlapping units of decision-making with their own jurisdictions, rules of access and use, monitoring and sanction systems, and conflict resolution mechanisms (Ostrom 1999, 2010). Polycentric governance differs from the notion of multilevel governance that assumes a hierarchical order between different governance units (Ros-Tonen et al. 2014).

Several cases reported in this special issue illustrate such tendency toward polycentrism in ILLIs. In Ghana, this encompasses a combination of statutory and customary institutions in the implementation of natural resource schemes (Foli et al. 2018). In China, state-controlled forest landscape restoration is gradually being transformed toward polycentric governance involving the engagement of the private sector and—be it still to a limited extent—civic actors as well as the incorporation of market-based policy instruments such as PES (Long et al. 2018, this issue).

Polycentric governance allows for flexibility, but also creates a coordination dilemma where one jurisdiction generates positive or negative externalities for other jurisdictions (Hooghe and Marks 2003). The paper by Dale et al. (2018) illustrates this well: in the Great Barrier Reef, landscape-level water-quality outcomes will not be automatically achieved based on the cumulative effect of fragmented governance subdomains that focus on different management problems (e.g., water allocation, land-use planning, indigenous management, farm-scale planning, or urban water management). The authors argue that a governance system analysis of how these subdomains perform against pre-defined design and evaluation principles for integrated landscape governance may create synergies that help prevent implementation failures in delivering water quality at the catchment level. Similarly, Rodríguez-Ward et al. (2018) point at the need for horizontal (multisectorial and multi-actor) and vertical (multilevel) coordination to achieve inclusive REDD+ governance at the landscape level. Both papers emphasize the role of the State in creating the conditions for effective steering and coordination. This is a shift away from neoliberal discourses that emphasize a retreating role of the State and a greater role for private actors and civil society (Jessop 2002; Bäckstrand and Lövbrand 2006). However Ndeinoma et al. (2018, this issue) note that civil society organizations may perform a similar role where they take on traditional government roles.

### ILLIs as Locally Embedded Entry Points for the Implementation of Integrated Landscape Approaches

The papers in this special issue evidence the increasing recognition that global challenges such as sustainable production and livelihoods, biodiversity conservation, and climate change mitigation need to be addressed in a holistic and integrated manner at the landscape level. However, the sectorial silos, particularly in the public sector, are not easily broken down (e.g., Dale et al. 2018; Foli et al. 2018; Ingram et al. 2018; Kusters et al. 2018; Long et al. 2018; Rodríguez-Ward et al. 2018). Institutional fragmentation and jurisdictional boundaries are major hindrances to implementing ILAs, and they hinder clarity about who is to steer such an approach or to implement its outcomes (van Oosten et al. 2013; Kusters et al. 2018, this issue). Moreover, there is a price tag to bringing different actors around the table; transaction costs are high (Sayer et al. 2015, 2016) and the financial capacity of local actors limited (Brown, 2018, this issue). In order to prevent donor dependency and ensure local buy-in of negotiated landscape solutions, it is therefore important to identify locally embedded entry points for the implementation of ILAs (Deans et al. 2018; Foli et al. 2018, this issue). The ILLIs brought together in this volume show mixed potential to function as such (Table 3). They all target multifunctional landscapes and multiple objectives, such as a combination of sustainable production and livelihoods with the preservation of other than provisioning ecosystem services such as water regulation (Dale et al. 2018; Eriksson et al. 2018; Long et al. 2018, this issue), biodiversity conservation (Deans et al. 2018; Ingram et al. 2018; van Oosten et al. 2018, this issue), cultural ecosystem services (van Oosten et al. 2018), and carbon sequestration (Foli et al. 2018; Rodríguez-Ward et al. 2018). They have also brought together actors from multiple sectors that typically did not collaborate prior to the initiative. This is particularly clear in the Cameroonian and Peruvian REDD+ cases (Brown 2018; Rodríguez-Ward et al. 2018, this issue) in which actors from the forestry and agricultural sectors take collective action for the first time.

**Table 3:** The potential and challenges of integrated landscape-level initiatives (ILLIs) as entry points for integrated landscape approaches

Type of ILLI^a^	Potential	Challenges	References^b^
Integrated forest and water management, Sweden	Integrated goals (economic, ecological, sociocultural); space for citizen and private sector participation; clear rules	Operationalization of how several objectives can best be balanced in an integrated approach	Eriksson et al. 2018
Forest landscape restoration, China	Integrated goals (economic, ecological, social); more equitable benefit sharing; new space for multi-stakeholder collaboration; greater fund-raising capacity	Non-participation of larger forest owners; limited civil society and community involvement; separation of land and tree ownership; political commitment with change in administration; hierarchical network dominated by the State	Long et al. 2018
Great Barrier Reef catchment management, Australia	Preventing implementation failure; improved coordination of governance in subdomains for increased water quality at catchment level	Institutional fragmentation; holistic catchment planning and coordination of subdomain governance; community commitment; power differences; and conflicting interests	Dale et al. 2018
Reforestation through co-management (MTS), Ghana	Integration of goals (forest restoration, securing future timber supply, livelihood improvement, carbon sequestration); multi-stakeholder design	Rigid, state-dominated decision-making structure; a lack of long-term economic incentives; broader partnerships dependent on donor funding; non-empowering capacity building; absence of monitoring and evaluation mechanisms	Foli et al. 2018
Community resource management areas (CREMAs), Ghana	Integrated approach, multi-stakeholder design for negotiation and collaboration; adaptive management; accommodates polycentric governance; capacity building	Limited scale and vertical connectedness; no monitoring and evaluation mechanisms in place	Foli et al. 2018
Chantier d’Aménagement, Burkina Faso	Potential for creating landscape-level synergies	Limited integration of development and conservation objectives; hierarchical governance structure; no dedicated platforms for stakeholder negotiation; non-empowering capacity building; no monitoring and evaluation mechanisms in place	Foli et al. 2018
REDD+, Peru	Creates new space for multilevel and multi-sector dialog and collaboration, notably between forestry, conservation and agriculture	Exclusion or marginal inclusion of key stakeholders; a lack of trust; conflicting interests; unequal power relations; weak governance structures; jurisdictional frictions; poor coordination	Rodríguez-Ward et al. 2018
Climate change mitigation, Cameroon	REDD+ as entry point for tackling livelihood and conservation concerns; WWF as “hybrid” that mobilizes funds and actors	Limited institutional (financial and human) capacity; restricted impact on livelihoods; short-term project funding	Brown 2018
Value chain governance for ecosystem services, The Netherlands	Awareness raising of need to preserve ecosystem services; multi-stakeholder involvement to address landscape-level issues (sourcing areas)	Integration of goals; sectorial focus; inclusiveness of the arrangements; adaptive learning approach; balancing state regulation and market governance; dealing with trade-offs	Ingram et al. 2018
Advanced value chain collaboration, Ghana	Sustainable cocoa production; enhanced natural, human, and social capital	Interventions at farm rather than landscape level; hierarchical relations; institutional and cultural rigidity; limited inclusiveness in decision-making; limited options for farmers’ self-organization; jurisdictional mismatches; and non-exclusion of relevant actors prevent negotiated decision-making on land use	Deans et al. 2018
Multifunctional oil palm concession, Indonesia	Place- and context-specific form of landscape governance; integration of production, environmental, social and cultural objectives	Sectorial focus; institutional rigidity and mismatch with existing legal frameworks; focus on concession rather than landscape level; resistance of central government against Zero Deforestation movement; reduced income from concession (but compensated through reduced social unrest)	Van Oosten et al. 2018
Multi-stakeholder platforms (piloted in Ghana and Indonesia)	Mobilizes landscape actors; enables multi-stakeholder negotiation and collaboration as well as monitoring and evaluation of landscape outcomes	High transaction costs; absence of good governance principles in government (notably transparency, legitimacy, and voice); power imbalances; inclusion of women; a lack of shared concern/sense of urgency; non-participation of key stakeholders (notably in Indonesia)	Kusters et al. 2018

^a^Lowore et al. and Ndeinoma et al. (this issue) have been excluded from this overview as they target products rather than landscapes;

^b^All references in this issue

Despite these encouraging signs, there are a number of other conditions that need to be fulfilled for ILLIs to evolve into integrated landscape approaches. These include, first, the need to create a sense of urgency of a commonly shared problem at the landscape level. As several cases have shown (e.g., Dale et al. 2018; Kusters et al. 2018), there is limited potential for collective action at the landscape level when such a sense of urgency is missing. Second, additional partnerships and alliances are needed to cover all jurisdictions within the landscape, including those of customary authorities (Foli et al. 2018; Deans et al. 2018) and indigenous rights holders (Rodríguez-Ward et al. 2018). Third, particular attention is needed to the inclusion of key stakeholders which might not be the primary stakeholders in the ILLI and are, therefore, excluded (e.g., the forestry agency and traditional land owners in the case of value chain collaboration (Deans et al. 2018) or key land users such as miners and farmers in REDD+ initiatives (Rodríguez-Ward, 2018). Attention to gender dynamics is a particular blind spot, poorly addressed in the papers of this special issue and the literature on landscape approaches in general (van Dijk and Bose 2016; Reed et al. 2016). Fourth, ILLIs (and ILAs for that matter) thrive better where principles of good governance are in place, as shown in the Sweden case (Eriksson et al. 2018) where forest tenure and other rules are clear and enforced, and there is ample space for citizen participation. Fifth, except in the paper by Eriksson et al. and van Oosten et al. (2018) we found limited evidence of attention to cultural ecosystem services in ILLIs such as cultural heritage and spiritual values, which are particularly important to local inhabitants. Sixth, some ILLIs with considerable potential for landscape governance require upscaling beyond the local level such as the CREMA case in Ghana (Foli et al. 2018). Creating a platform to negotiate and monitor objectives at a landscape level can be a means of doing so (Ros-Tonen et al. 2015a; Sayer et al. 2016; Foli et al. 2018; Kusters et al. 2018, this issue).

### A Framework to Assess and Monitor Performance of Integrated Approaches

Participatory monitoring of progress and outcomes of landscape approaches and landscape-level initiatives is key to adaptive management and the capacity to deal with the dynamics inherent in landscapes, but data collection is often costly and time-consuming (Sayer et al. 2013; 2015). EcoAgriculture Partners (2017) developed a simple scorecard to assess the institutional capacity and performance of actors active in integrated landscape governance, an adapted version of which was applied by Brown (2018, this issue) in Cameroon to identify strengths and weaknesses in financial and human capacity, longevity in the landscape, demonstrated leadership, coordination with other organizations, and influence. The participatory monitoring and evaluation (PME) method proposed by Kusters et al. (2018, this issue) not only looks at the performance of the multi-stakeholder process (“looking inward” in terms of good governance principles and conditions for effective multi-sector collaboration), but also helps them in “looking ahead” (joint priority setting regarding conservation, production, and production objectives and institutional strengthening) and “looking back” (evaluating the outcomes and whether platform objectives have been met). Pilots in Ghana and Indonesia illustrated that such a method can work well if relevant stakeholders are committed to participate in the PME workshop; reflect critically on all the actors, including themselves; and follow-up on the recommendations that result from the process. Such a method could be particularly useful for cases where concrete monitoring and evaluation mechanisms are missing, as in the natural resource management schemes analyzed in Foli et al. (2018, this issue)—particularly if such schemes are a response to failures of past initiatives. Moreover, monitoring and evaluation is key to dealing with landscape-level dynamics, uncertainty, and complexity, and fundamental to the application of adaptive management (Sayer et al. 2016).

## Conclusion

This special issue illustrates a trend toward greater synergies in objectives and actor configurations in natural resource management and value chain governance. Starting with product-focused approaches in the NTFP sector aiming at combined development and conservation outcomes, the current trend is toward landscape-level initiatives and integrated landscape approaches that consider and negotiate trade-offs between different land uses and objectives. Despite design principles that suggest a common umbrella (Sayer et al. 2013; Ros-Tonen et al. 2014), landscape approaches are, to paraphrase Max Eggert (2015), like jellyfish: an increasing scholarship knows what they are, but there are no two people who understand them in a similar manner or even use the same term (Scherr et al. 2013; Reed et al. 2016). As landscapes are in the eyes of the beholder (*c.f.* Meinig 1979), integrated landscape approaches and landscape-level initiatives come in different sizes and shapes. This review and the papers in this special issue make clear that it is useful to distinguish between ILAs, which are designed as negotiated landscape governance from the beginning, and ILLIs that started as sectorial approaches but are moving toward more integrated multi-stakeholder approaches targeting multifunctional landscapes. The tendency toward integrated landscape approaches is growing, but balancing multiple objectives, equitable inclusion of all relevant stakeholders, dealing with power and gender imbalances, adaptive management based on participatory outcome monitoring, and moving beyond existing administrative, jurisdictional, and sectorial silos remain significant challenges. Overall, we conclude that, first, actors can only be mobilized around a commonly felt problem and sense of urgency, and that landscape approaches are therefore necessarily context- and issue-specific. Second, dealing with multiple objectives, trade-offs, as well as with power imbalances and conflicting interests, implies a key role for multi-stakeholder platforms, bridging organizations and “watchdogs” to negotiate priorities and give voice to weaker parties. Third, considering the challenges that integrated landscape approaches are still facing, ILLIs may be feasible and locally embedded entry points to their implementation. Fourth, the multilayered nature of human–nature interactions at the landscape level—vertical (multilevel), horizontal and cross-cutting (“zigzagging”; Torfing et al. 2012)—requires accommodating multiple centers of decision-making in fluid, polycentric governance arrangements. This involves statutory and customary as well as public and private institutions, but requires an overseeing actor—government or bridging organization—who can steer the process. Last, but not least, implementing integrated landscape approaches implies the need to deal with diversity and dynamics. Such challenges suggest that an element of “muddling through” (Lindblom 1959; Sayer et al. 2008; Colfer et al. 2011) will be necessary for landscape approaches to evolve. However, “muddling through” need not imply muddled thinking or a state of imperfection, but is inherent in dealing with landscapes.

## Electronic supplementary material

Supplementary Material

## Footnotes

1 We take ILAs and ILIs as being synonymous and use ILAs in the rest of this paper.

2 Including, among others, the Centre of International Forestry Research, CIFOR (Frost et al. 2006; Sunderland et al. 2008; Reed et al. 2016, 2017), EcoAgrculture Partners (Scherr et al. 2013; Kozar et al. 2014), the Global Landscapes Forum (GLF.org), the Food and Agriculture Organization of the United Nations (FAO 2016), ICRAF, the World Agroforestry Centre (Minang et al. 2015), the International Union for the Conservation of Nature (IUCN) (Brown et al. 2005), Tropenbos International (Kusters et al. 2016), the World Bank (World Bank Group 2016), and the World Wide Fund for Nature (WWF 2002).

3 Scales are understood as the spatial, temporal, jurisdictional or institutional dimensions of a phenomenon under study; levels to different positions on a scale (Gibson et al. 2000; Cash et al. 2006). In this conceptualization, a landscape is a level on a spatial or geographical scale (Cash et al. 2006).

4 The cases reported by Lowore et al. and Ndeinoma et al. (2018) are excluded from this count as they have a sectorial focus; the case reported by Kusters et al. was excluded because it concerns a pilot to test a methodology for an ILA.

5 Of the 24 ILLIs, 8 were initiated by the private sector and 2 through a public–private partnership. Ten of the remaining cases were initiated by the public sector and 4 by a civil society organization with or without a university or knowledge institution. See supplementary material 1.^.^
