# Supplementary material for: From Synergy to Complexity: The Trend Toward Integrated Value Chain and Landscape Governance
Source: Environ Manage. 2018 May 30;62(1):1–14. doi: 10.1007/s00267-018-1055-0 (PMC5999153; doi:10.1007/s00267-018-1055-0)
Supplement: Supplementary file 1 — Supplementary Material [file 267_2018_1055_MOESM1_ESM.docx]

**Supplementary material 1.** Actor constellations in the cases discussed in this special issue

| Country / Case | Initiator |  | | Actors involved^a^ | | | | |  | Reference |
| --- | --- | --- | --- | --- | --- | --- | --- | --- | --- | --- |
|  |  | Public | Private | Small-scale producers/  communities | Civic | | University/  knowledge institutions | Donors | Certification organization |  |
| *Ethiopia* |  |  |  |  |  | |  |  |  |  |
| Forest honey^b^ | University of  Huddersfield | √ |  | √ | **√** | | **√** | √ |  | Wood and Bekele Haile 2009; Lowore et al., this issue |
| *Namibia* |  |  |  |  |  | |  |  |  |  |
| Indigenous Natural Plants (several governance bodies) | Ministry of Environment and Tourism (MET); Ministry of Agriculture, Water and Forestry | **√** | (√)^c^ | (√)^d^ | √ | |  |  |  | Ndeinoma et al., this issue |
| *Sweden* |  |  |  |  |  | |  |  |  |  |
| Integrated forest and water management Sweden (several cases) | Swedish government (through Forest Act and Forest Agency) | **√** | √ |  | √ | |  |  |  | Eriksson et al., this issue |
|  | Forest certification organizations |  | √ |  |  | |  |  | **√** |  |
| “Water in Forests” | Forest owner association and WWF |  | **√** |  | √ | |  | √ |  |  |
| “Blue Targeting” | Forest owner association and WWF |  | **√** |  | √ | | √ | √ |  |  |
| EU Water Framework Directive | EU | **√** | √ |  |  | |  |  |  |  |
| *China* |  |  |  |  |  | |  |  |  |  |
| EFPP (forest restoration) | Yong’an municipality gov’t | **√** | √ | √ | (√)^e^ | |  |  |  | Long et al., this issue |
| *Australia* |  |  |  |  |  | |  |  |  |  |
| GBR governance | Queensland gov’t | **√** | √ | √ | √ | |  |  |  | Dale et al., this issue |
| *Burkina Faso* |  |  |  |  |  | |  |  |  |  |
| Chantier d’Aménagment Forestier | MEDD | **√** |  | √ | (√) | |  | (√) |  | Foli et al., this issue |
| *Peru* |  |  |  |  |  | |  |  |  |  |
| REDD+ | REDD+ Roundtable Peru, WWF | √ | √ | (√) | **√** | | (√) | √ |  | Rodríguez-Ward et al., this issue |
| *Cameroon* |  |  |  |  |  | |  |  |  |  |
| REDD+ | WWF | √ | √ | (√) | **√** | | √ | √ |  | Brown, this issue |
| *The Netherlands* |  |  |  |  | |  |  |  |  |  |
| Value chain governance for environmental services (9 cases) | Dutch gov’t | **√** | √ |  | |  |  |  |  | Ingram et al., this issue |
| - STAP 2010 (cocoa) & Utz certification 2008 | IDH | √ | **√** | (√)^d^ | | √ | (√)^f^ |  | √ |  |
| - PES 2010 (cocoa) | Cocoa companies | **√** | **√** | (√)^d^ | | √ |  |  |  |  |
| - RTRS 2006 (soy) | Soy companies | √ | **√** |  | | √ | (√)^f^ |  | √ |  |
| - RSPO 2003 (oil palm) | Oil palm companies | √ | **√** |  | | √ | (√)^f^ |  | √ |  |
| - STAP 2011 (timber) | Dutch gov’t | **√** | √ |  | | √ | (√)^f^ |  | √ |  |
| - FSC 1993 & ForCES 2012 (timber) | Forest owners, concessionaires, timber industry | √ | **√** |  | | √ | (√) |  | √ |  |
| - Dutch Public Procurement Policy 2008 (timber) | Dutch gov’t | **√** | √ |  | | (√) |  |  | √ |  |
| - REDD+ Indonesia 2010 (timber) | Coalition of NGOs, UN, Dutch gov’t | **√** | **√** | (√) | | **√** | (√) | √ |  |  |
| *Ghana* |  |  |  |  | |  |  |  |  |  |
| MTS (reforestation) | FC (Forest Services Division) | **√** | √ |  | | (√)^g^ |  | (√)^g^ |  | Foli et al., this issue |
| CREMA (resource management) | FC (Wildlife Division) | **√** |  |  | | (√)^d^ |  | (√)^h^ |  |  |
| Advanced VCC | AGL Ltd. | (√)^i^ | **√** | √ | |  |  |  | √ | Deans et al., this issue |
| Multi-stakeholder platform (landscape governance) | Tropenbos International/EcoAgriculture Partners | √ | √ | √ | | **√** | **√** | √ |  | Kusters et al., this issue |
| *Indonesia* |  |  |  |  | |  |  |  |  |  |
| PPP in oil-palm landscape | IOPG/Oil palm company | √ | **√** | √ | |  |  |  |  | Van Oosten et al., this issue |
| Multi-stakeholder platform (landscape governance) | Tropenbos International/EcoAgriculture Partners | √ | √ | √ | | **√** | **√** | (√) |  | Kusters et al., this issue |
|  |  |  |  |  | |  |  |  |  |  |
|  |  |  |  |  | |  |  |  |  |  |

*Key:* AGL = Armajaro Ghana Limited (cocoa); CREMA = Community Resource Management project; EFPP = Ecological Forest Purchase Program (China); EU = European Union; FC = Forestry Commission (Ghana); ForCES = Forest Certification for Ecosystem Services; FSC = Forest Stewardship Council; GBR = Great Barrier Reef; IDH = Initiatief Duurzame Handel (Sustainable Trade Initiative); IOPG = Indonesian Palm Oil Innovation Group; MEDD = Ministry of Forest, Environment and Sustainable Development (Burkina Faso); MTS = Modified taungya system (reforestation scheme, Ghana); PBC = Produce Buying Company (cocoa, Ghana); PES = Payments for Environmental Services; PPP = public-private partnership; REDD+ = Reducing Emissions from Deforestation and forest Degradation plus; RSPO = Roundtable for Sustainable Palm Oil; RTRS = Roundtable for Responsible Soy; STAP = Sustainable Trade Action Plan (NL); UN = United Nations; WWF = World Wide Fund for nature

^a^ In bold: initiating sector; (√) = indirectly or marginally involved.

^b^ Information retrieved from the project’s progress report 2008-2009 (Wood and Bekele Haile 2009).

^c^ Mainly consultants.

^d^ Indirect as target groups.

^e^ Government-led NGO.

^f^ Indirect via monitoring and evaluation.

^g^ Donor funding from the African Development Bank from 2008-2012. During that time civil society organizations (CSOs) were also involved.

^h^ CREMA is a nationally embedded initiative initiated by the Wildlife Division of the Ghana Forestry Commission, independent of CSO and donor involvement, but may locally enjoy funding by IUCN and UNDP, among others.

^i^ Public sector involved through the Ghana Cocoa Board (COCOBOD) as buyer of cocoa, but not as direct partner in the initiative.
